# Truncal Changes in Patients After Total Hip or Knee Arthroplasty: A Surface Topography Study

**DOI:** 10.7759/cureus.4260

**Published:** 2019-03-18

**Authors:** Vasileios A Kechagias, Theodoros BBBB Grivas, Panagiotis J Papagelopoulos, Vasileios A Kontogeorgakos, Konstantinos Vlasis

**Affiliations:** 1 Orthopaedics, General Hospital of Volos, Volos, GRC; 2 Orthopaedics and Traumatology, Tzaneio General Hospital of Piraeus, Piraeus, GRC; 3 Orthopedics, ATTIKO - General University Hospital, Athens, GRC; 4 Orthopaedics, Athens University Medical School, Athens, GRC

**Keywords:** trunkal changes, total hip arthroplasty, total knee arthroplasty, surface topography

## Abstract

Introduction

Total hip and knee arthroplasty (THA/TKA) are among the most successful orthopedic operations performed, aiming at the alleviation of osteoarthritic pain. This report is a surface topography study that assesses the mechanism by which THA or TKA influences truncal parameters. This study represents the first time that surface topography has been used for the assessment of truncal parameters.

Methods

In total, 15 patients with THA, including nine women and six men, with a mean age of 65.07 ± 9.73 years (range: 47-80 years), and 23 patients with TKA were assessed preoperatively and four and 12 months postoperatively. These patients were also compared with a control group (CG) that included 25 individuals, including 12 women and 13 men, with a mean age of 69.28 ± 10.11 years (range: 55-86 years). The Diers Formetric four-D analysis system was used to calculate several truncal parameters in all planes.

Results

Data analysis revealed statistically significant differences in the kyphotic angle (°; 56.50→63.57, *p *< 0.05) and the pelvic obliquity (°; 3.40→1.93, *p *< 0.05) between measurements at baseline and 12 months after THA. Statistically significant differences were noted for the pelvic obliquity (°; 2.74→1.43, *p *< 0.05) between measurements at baseline and 12 months after TKA.

Conclusions

THA and TKA are causes of truncal morphological alterations. THA and TKA improved pelvic obliquity, contributing to correct the posture in the patients.

## Introduction

Osteoarthritis (OA) is a major public health problem worldwide that typically affects the hip and knee joints [1]. The lifetime risk for symptomatic hip OA is 18.5% for men and 28.6% for women [2]. Symptomatic knee OA happens in 10% of men and 13% of women aged 60 years or older [3]. Hip and knee OA can lead to marked disability, pain, decreased mobility, and the need for increased usage of healthcare services. Total hip or knee arthroplasty (THA/TKA) is the gold standard treatment for painful hip and knee OA. One million THA procedures are performed each year worldwide [4]. TKA is the most commonly performed surgical procedure in the USA, with an estimated 3.48 million procedures performed annually by 2030 [5]. THA or TKA not only restores joint motion but also relieves pain due to OA.

There is a scarcity of studies on how THA or TKA influences the parameters of the spine and the pelvis. Normal alignment of the spine and pelvis in the sagittal plane is critical to maintain an upright standing position with minimal energy consumption [6-7]. The objectives of this study are to investigate changes in the truncal parameters after THA or TKA and assess whether these parameters are improved or deteriorated after these operations. Ιt is also worth mentioning that this study represents the first time that surface topography has been used for the assessment of truncal parameters. Surface topography is a non-invasive and radiation-free method compared with radiographs and is a reliable method for three-dimensional back shape imaging [8-11].

## Materials and methods

Overview

This is a prospective study of the effect of THA or TKA upon truncal morphological parameters in patients who underwent an operation for severe hip or knee OA between April 2014 and March 2016. Four orthopedic surgeons carried out THA and TKA. This study was approved by an institutional review board, and all the patients consented. Patients with a diagnosis of unilateral hip or knee OA who elected to undergo THA or TKA were recruited and assessed. The following exclusion criteria were applied: (1) marked OA in other joints of lower extremities, (2) arthritis secondary to other diseases, e.g., ankylosing spondylitis, rheumatoid arthritis, developmental dysplasia, and trauma, (3) neurological deficits in lower extremities, (4) history of surgical intervention in the spine or lower extremities, and (5) other diseases that would affect the alignment of the trunk. All the patients were examined for truncal parameters using the Diers Formetric four-D analysis system preoperatively and at four months and one year postoperatively. The patients were almost fully recovered with almost full range of motion four months postoperatively. After four months, rehabilitation programmes were typically stopped. Between the fourth and 12th months; further improvement in the outcomes was noted in the operated patients. Finally, 12 months postoperatively, the patients were fully recovered with a full range of motion and no pain. Patients returned to their normal activities at this time point [1-2]. Therefore, the influence of THA or TKA upon truncal parameters was initially (fourth month) and finally (12th month) assessed in fully recovered patients. In addition, a control group (CG) was assessed for truncal parameters using the Diers Formetric four-D analysis system for comparison with patients with THA or TKA. Finally, secure and precise results regarding the effect of THA or TKA upon the truncal parameters and whether these changes were similar to the normal levels of the CG were obtained.

Patients with THA

Initially, 20 patients were recruited into the prospective study. However, four patients refused to undergo follow-up, and one patient had a pulmonary embolism. In total, 15 patients, including nine women and six men, with a mean age of 65.07 ± 9.73 years (range, 47-80 years) were assessed. All the patients were assessed preoperatively and four and 12 months postoperatively. Eight patients underwent an operation for the right hip and seven for the left hip. Other demographic characteristics for these patients include a mean weight of 81.67 ± 17.99 kg, mean height of 166.47 ± 10.25 mm, and mean body mass index (BMI) of 29.26 ± 4.14 kg/m^2^.

Patients with TKA

Initially, 31 patients were recruited into the prospective study. However, seven patients refused to undergo follow-up, and one patient experienced a hip fracture four months postoperatively. In total 23 patients, including 19 women and four men, with a mean age of 71.48 ± 8.09 years (range, 54-90 years) were assessed. All the patients were assessed preoperatively and four and 12 months postoperatively. Thirteen patients underwent an operation for the right knee and 10 for the left knee. Other demographic characteristics for these patients included a mean weight of 77.35 ± 12.75 kg, mean height of 160.83 ± 6.09 mm, and mean BMI of 30.04 ± 4.67 kg/m^2^.

Control group 

This group consisted of 25 individuals, including 12 women and 13 men, with a mean age of 69.28 ± 10.11 years (range, 55-86 years). Other demographic characteristics for these normal individuals included a mean weight of 79.40 ± 13.08 kg, mean height of 165.04 ± 9.46 mm, and mean BMI of 29.00 ± 3.00 kg/m^2^. These persons were matched closely with the patients with THA or TKA, and no statistical differences in demographic variables were noted between patients and CG. The following inclusion criteria were applied to the CG: (1) no OA of the hip or knee joints, (2) no arthritis secondary to other diseases, (3) no neurological deficit n the lower extremity, (4) no history of surgical intervention in the spine or lower extremity, and (5) no dieases that would affect the alignment of the trunk.

The apparatus and the studied parameters

**Figure 1:**
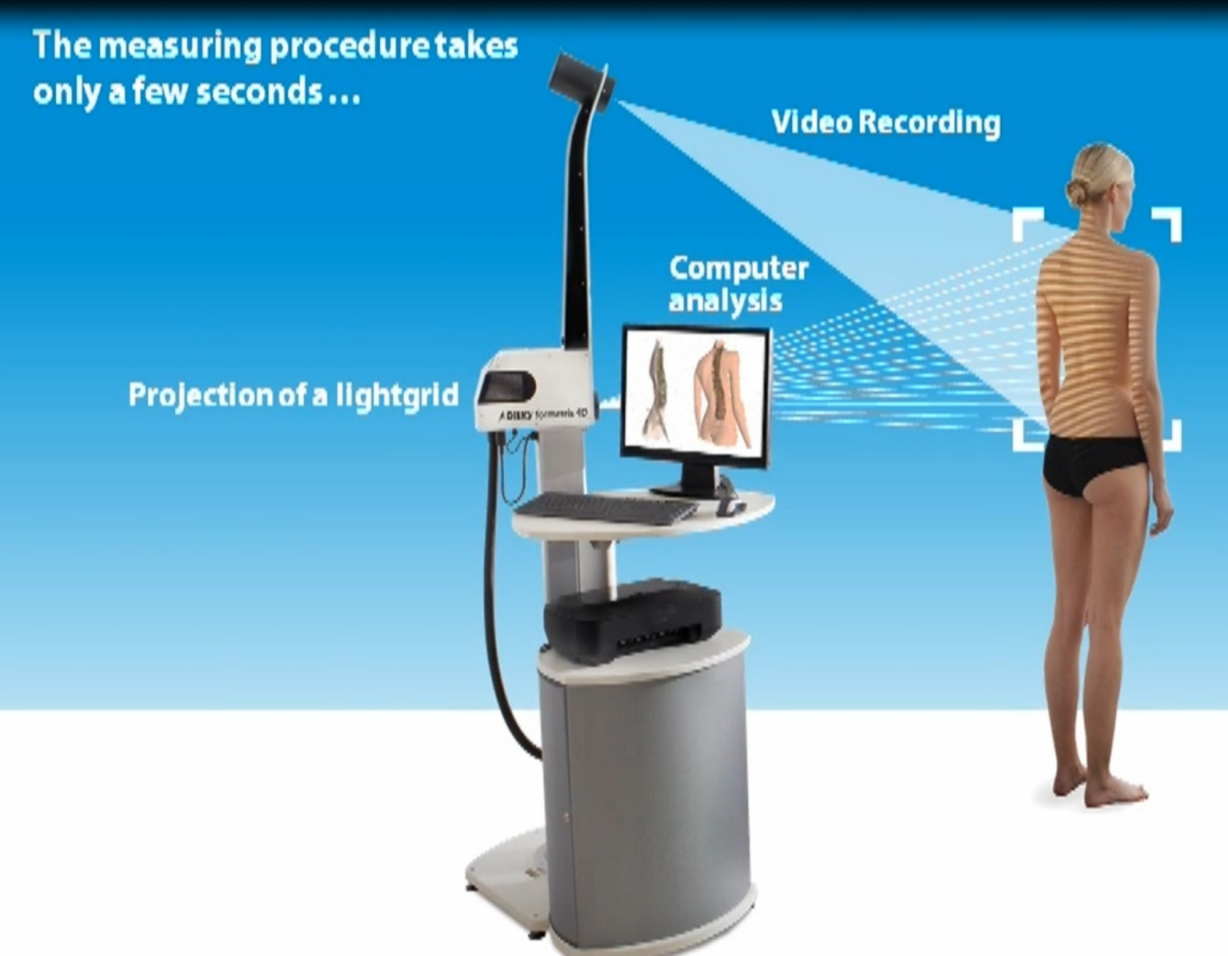
The Diers Formetric four-D analysis system function (used with permission from DIERS)

The Diers Formetric four-D analysis system was used for photogrammetric recording of the back using a video raster stereography process [8-11]. This system is one surface topography instrument, which combines rasterstereography and biomechanical modeling to obtain body surface measurements. This technique projects horizontal stripes of light onto the surface of the patient's back, and static images of the lines are recorded and digitized. Based on the distortion a three-dimensional image of the back can be produced and measured. So the patients stood approximately 2 m in front of the system. A light grid was projected on the patientʼs back trunk surface, and the system recorded the concave and convex areas of the truncal surface (Figure 1).

**Figure 2:**
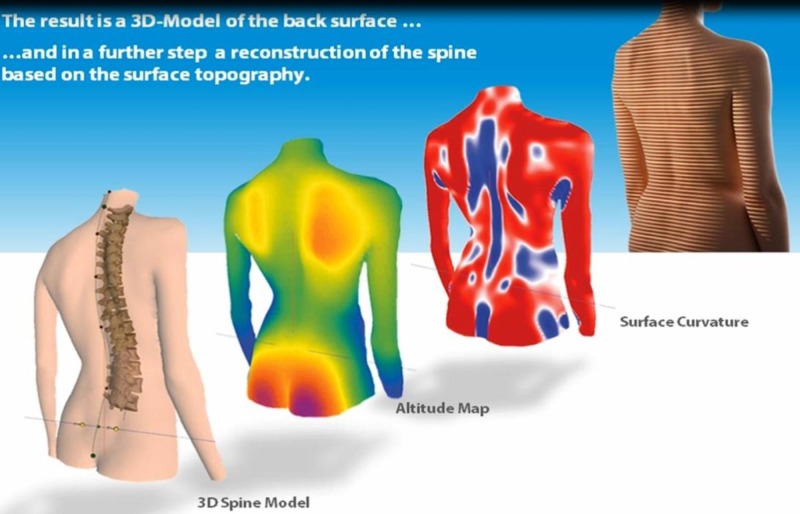
The Diers Formetric four-D analysis system result (used with permission from DIERS)

Anatomical landmarks were distinguished automatically by the system. The system measured patients over a six-second period of time, taking two pictures per second. The 12 acquired images were assessed by the system’s software, correcting any subject movement. Finally, utilizing a complex algorithm, the data results provided an accurate three-dimensional model (coronal, sagittal, transverse plane) of the back surface and a reconstruction of the spinal column and pelvis based on the surface topography (Figure 2).

**Figure 3:**
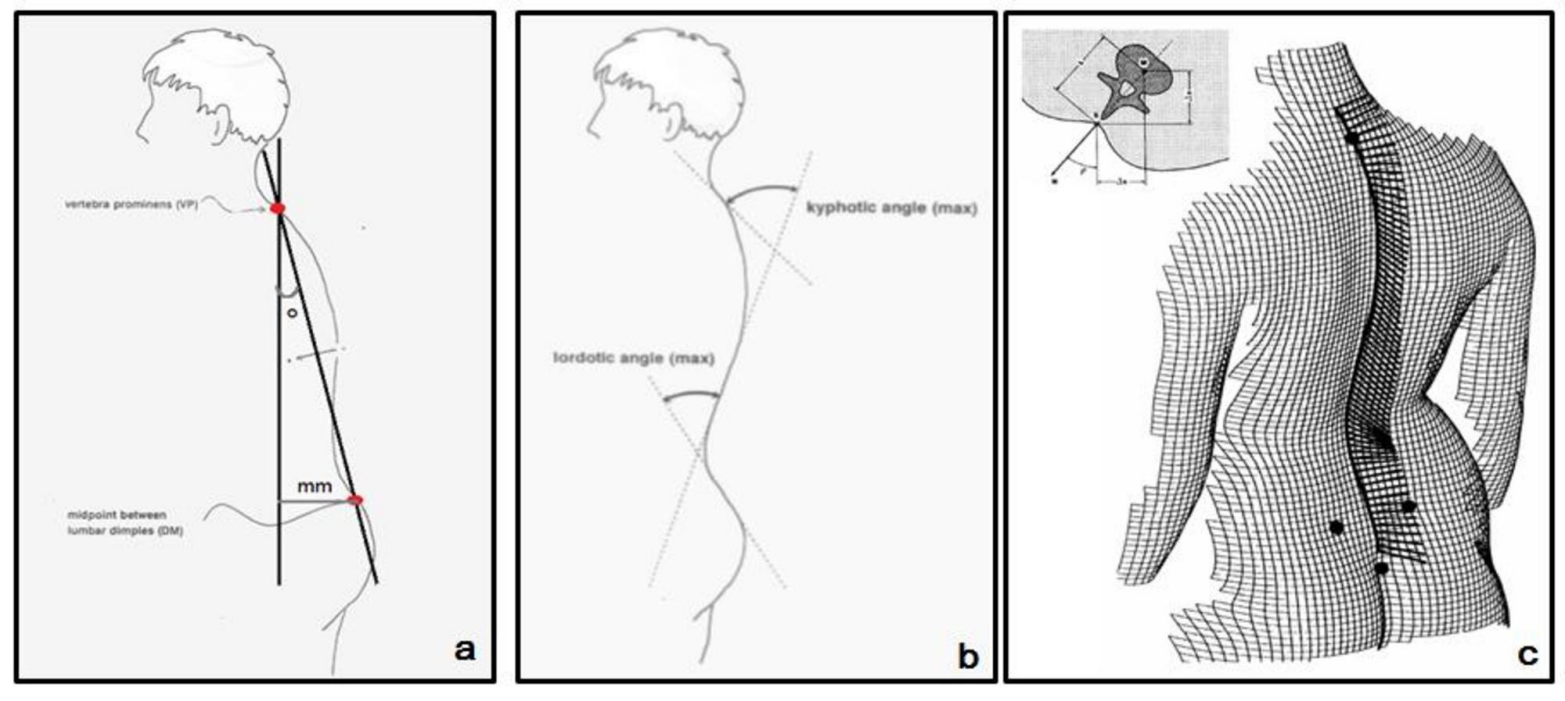
Illustration of the sagittal imbalance (a), the lordotic and the kyphotic angle (b), the vertebral rotation and trunk torsion (c)

Several parameters were calculated from the system [10]. The following parameters were assessed in the spine (Figure 3): (1) coronal plane: the coronal imbalance, the apical deviation and the scoliosis angle, (2) sagittal plane: the kyphotic angle, the lordotic angle and the sagittal imbalance, (3) transverse plane: the vertebral rotation and the trunk torsion.

**Figure 4:**
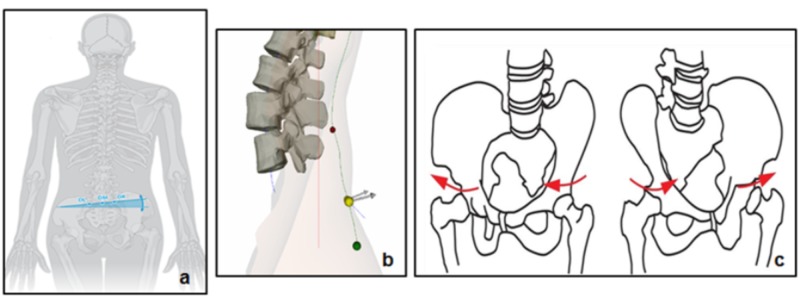
Illustration of pelvic obliquity (a), pelvic inclination (b) and pelvis rotation (c)

The following parameters were assessed in the pelvis (Figure 4): (1) coronal plane: the pelvic obliquity, (2) sagittal plane: the pelvic inclination and the pelvic torsion, (3) transverse plane: the pelvis rotation.

The Diers Formetric four-D analysis system is a non-contact, automatic measurement system that does not use radiation. The main purpose is to capture shape parameters to establish the body posture and to calculate the shape of the spinal column, back surface, and pelvic position. The measurements are very reproducible with high validity and accuracy compared with X-rays [8-11].

Statistical analysis

Data were expressed as mean±Standard Deviation (SD) for quantitative variables and percentages for qualitative variables. The Kolmogorov-Smirnov test was utilized for normality analysis of quantitative variables. The homogeneity of compared groups in relation to demographic characteristics was examined using the Independent samples t-test and Fisher’s exact test. The comparison of variables at each time point between the CG and operated populations was performed using the Independent samples t-test or Mann-Whitney test in case of violation of normality. A one-factor repeated measures analysis of variance (ANOVA) model was used for the comparison of different time measurements of variables for the operated group. Pairwise multiple comparisons were performed applying the Bonferroni test. Friedman and Wilcoxon tests were used in case of violation of normality. All tests were two-sided, and a *p*-value of <0.05 was used to denote statistical significance. All analyses were achieved using the Statistical Package for the Social Sciences (SPSS) version 17.00 (Chicago, III, United States of America).

## Results

The homogeneity of demographic characteristics between the CG and the patients with THA and TKA is summarized in Table 1. No statistically significant differences were noted between the CG and patients with THA for all demographic variables, including age (*p *= 0.204), gender (*p* = 0.527), weight (*p* = 0.648), height (*p *= 0.657), and BMI (*p *= 0.822). No statistically significant differences were noted between the CG and patients with TKA for all demographic variables, including age (*p* = 0.412), weight (*p *= 0.585), height (*p *= 0.076), and BMI (*p *= 0.363) except for gender (*p *= 0.017).

**Table 1:** Homogeneity of demographic characteristics between the CG and patients with THA and TKA CG: control group; THA: total hip arthroplasty; TKA: total knee arthroplasty; SD: standard deviation; BMI: body mass index

	Demographic characteristics	Control Group	Patients	p-value
		CG ( n=25 )	Patients ( n=15 )	p-value
THA	Age, Mean±SD	69.28±10.11	65.07±9.73	0.204
	Gender, male/female n (%)	13(52%)/12(48%)	6(40%)/9(60%)	0.527
	Weight, Mean±SD	79.40±13.08	81.67±17.99	0.648
	Height, Mean±SD	165.04±9.46	166.47±10.25	0.657
	ΒΜI, Mean±SD	29.00±3.00	29.26±4.14	0.822
	Operated leg (right/left) n (%)	---	8(53.3%)/7(46.7%)	---
		CG ( n=25 )	Patients ( n=23 )	p-value
TKA	Age, Mean±SD	69.28±10.11	71.48±8.09	0.412
	Gender, male/female n (%)	13(52%)/12(48%)	4(17.4%)/19(82.6%)	0.017
	Weight, Mean±SD	79.40±13.08	77.35±12.75	0.585
	Height, Mean±SD	165.04±9.46	160.83±6.09	0.076
	ΒΜΙ, Mean±SD	29.00±3.00	30.04±4.67	0.363
	Operated leg (right/left) n (%)	---	13(56.5 %)/10(43.5%)	---

Patients with THA

The parameters of the spine and the pelvis in the patients assessed both preoperatively and four and 12 months after THA are summarized in Table 2. No statistically significant differences were noted between time measurements for all parameters except kyphotic angle (°; *p *= 0.018) and pelvic obliquity (°; *p* = 0.030). Pairwise comparisons indicated statistically significant differences between measurements at baseline and 12 months postoperatively for the kyphotic angle (°; 56.50→63.57, *p *< 0.05) and the pelvic obliquity (°; 3.40→1.93, *p *< 0.05).

**Table 2:** Comparison of parameters for patients with THA during the observation period *p < 0.05 vs baseline THA, total hip arthroplasty; SD, standard deviation; rms, root mean square

	THΑ baseline		THΑ 4 months		THΑ 12 months		Overall p-value
	mean	SD	mean	SD	mean	SD	
kyphotic angle (°)	56.50	13.53	60.41	11.63	63.57*	10.10	0.018
lordotic angle (°)	48.53	12.27	45.74	12.31	47.80	12.19	0.417
sagittal imbalance (°)	9.47	5.12	8.11	4.70	8.96	4.90	0.434
coronal imbalance (°)	1.27	1.03	1.47	1.46	1.60	1.30	0.403
apical deviation rms (mm)	6.59	3.87	5.91	2.78	6.52	2.96	0.744
scoliosis angle (°)	16.80	9.06	15.27	7.83	16.80	8.42	0.474
vertebral rotation rms (mm)	5.07	3.04	4.15	1.71	4.85	2.51	0.781
trunk torsion (°)	8.53	7.19	5.47	5.64	7.67	5.09	0.213
pelvic inclination (°)	26.07	9.60	24.71	7.97	25.21	8.51	0.546
pelvic obliquity (°)	3.40	2.44	2.47	2.00	1.93*	2.40	0.030
pelvic torsion (°)	3.60	1.84	2.93	1.53	3.40	1.99	0.689
pelvis rotation (°)	3.40	4.48	0.73	1.83	1.33	1.63	0.074

The comparisons between the CG and the patients with THA during the observation period are summarized in Table 3. Preoperatively, patients presented with significantly increased values compared with the CG for sagittal imbalance (°; 4.27→9.47, *p* = 0.002), trunk torsion (°; 4.16→8.53, *p* = 0.042), pelvic inclination (°; 19.04→26.07, *p* = 0.028), and pelvic obliquity (°; 0.96→3.40, *p *= 0.002). At four months postoperatively, patients presented statistically significant increased values compared with the CG for sagittal imbalance (°; 4.27→8.11, *p *= 0.009) and pelvic obliquity (°; 0.96→2.47, *p *= 0.004). At 12 months postoperatively, the patients exhibited significantly increased values compared with the CG for kyphotic angle (°; 56.18→63.57, *p *= 0.024), sagittal imbalance (°; 4.27→8.96, *p* = 0.003), trunk torsion (°; 4.16→7.67, *p *= 0.018), and pelvic inclination (°; 19.04→25.21, *p *= 0.043).

**Table 3:** Comparison between the CG and patients with THA group during the observation period CG, control group; THA, total hip arthroplasty, SD, standard deviation; rms, root mean square

	CG		THΑ baseline		p-value baseline	THΑ 4 months		p-value 4 months	THΑ 12 months		p-value 12 months
	mean	SD	mean	SD		mean	SD		mean	SD	
kyphotic angle (°)	56.18	9.37	56.50	13.53	0.930	60.41	11.63	0.214	63.57	10.10	0.024
lordotic angle (°)	42.26	9.13	48.53	12.27	0.072	45.74	12.31	0.312	47.80	12.19	0.110
sagittal imbalance (°)	4.27	2.70	9.47	5.12	0.002	8.11	4.70	0.009	8.96	4,90	0.003
coronal imbalance (°)	1.16	0.69	1.27	1.03	0.726	1.47	1.46	0.454	1.60	1.30	0.240
apical deviation rms (mm)	4.94	1.81	6.59	3.87	0.137	5.91	2.78	0.187	6.52	2.96	0.076
scoliosis angle (°)	12.96	4.45	16.80	9.06	0.142	15.27	7.83	0.241	16.80	8.42	0.066
vertebral rotation rms (mm)	3.98	1.65	5.07	3.04	0.145	4.15	1.71	0.756	4.85	2.51	0.189
trunk torsion (°)	4.16	3.82	8.53	7.19	0.042	5.47	5.64	0.387	7.67	5.09	0.018
pelvic inclination (°)	19.04	8.97	26.07	9.60	0.028	24.71	7.97	0.056	25.21	8.51	0.043
pelvic obliquity (°)	0.96	1.10	3.40	2.44	0.002	2.47	2.00	0.004	1.93	2.40	0.157
pelvic torsion (°)	2.64	1.50	3.60	1.84	0.080	2.93	1.53	0.556	3.40	1.99	0.178
pelvis rotation (°)	2.08	2.48	3.40	4.48	0.308	0.73	1.83	0.057	1.33	1.63	0.307

Patients with TKA

The parameters of the spine and the pelvis in the patients assessed preoperatively and four and 12 months after TKA are summarized in Table 4. No statistically significant differences were noted between time measurements for all parameters except pelvic obliquity (*p *= 0.021). Pairwise comparisons indicated statistically significant differences between baseline and 12 months postoperatively for pelvic obliquity (°; 2.74→1.43, *p *< 0.05).

**Table 4:** Comparison of parameters for patients with TKA during the observation period *p < 0.05 vs baseline TKA, total knee arthroplasty; SD, standard deviation; rms, root mean square

	TKΑ baseline		TKΑ 4 months		TKΑ 12 months		Overall p-value
	mean	SD	mean	SD	mean	SD	
kyphotic angle (°)	62.46	13.70	63.15	15.11	63.67	12.51	0.498
lordotic angle (°)	48.76	16.80	45.46	14.98	48.30	17.02	0.623
sagittal imbalance (°)	7.57	4.46	7.89	3.93	7.70	4.67	0.838
coronal imbalance (°)	1.22	1.00	1.17	1.03	1.35	1.11	0.710
apical deviation rms (mm)	5.94	2.70	6.05	2.58	5.31	2.27	0.344
scoliosis angle (°)	16.39	5.81	17.70	5.88	15.13	4.91	0.301
vertebral rotation rms (mm)	4.52	2.58	5.57	3.10	5.14	2.57	0.332
trunk torsion (°)	7.65	6.46	8.04	4.78	7.70	6.03	0.965
pelvic inclination (°)	25.43	13.04	23.17	11.96	24.74	12.98	0.522
pelvic obliquity (°)	2.74	2.78	1.83	2.44	1.43^*^	1.73	0.021
pelvic torsion (°)	2.57	2.11	2.43	1.75	2.04	1.46	0.283
pelvis rotation (°)	3.78	4.80	2.17	3.10	1.61	2.35	0.061

The comparison between the CG and the patients with TKA during the observation period are summarized in Table 5. Preoperatively, patients presented with significantly increased values compared with the CG for sagittal imbalance (°; 4.27→7.57, *p *= 0.004), scoliosis angle (°; 12.96→16.39, *p *= 0.026), trunk torsion (°; 4.16→7.65, *p *= 0.031), and pelvic obliquity (°; 0.96→2.74, *p *= 0.008). At four months postoperatively, the patients continued to present significantly increased values compared with the CG for sagittal imbalance (°; 4.27→7.89, *p *= 0.001), scoliosis angle (°; 12.96→17.70, *p *= 0.003), vertebral rotation root mean square (rms, mm; 3.98→5.57, *p *= 0.036), and trunk torsion (°; 4.16→8.04, *p *= 0.003). At 12 months postoperatively, patients presented with significantly increased values compared with the CG for kyphotic angle (°; 56.18→63.67, *p *= 0.023), sagittal imbalance (°; 4.27→7.70, *p *= 0.004), and trunk torsion (°; 4.16→7.70, *p *= 0.018).

**Table 5:** Comparison between the CG and patients with TKA group during the observation period CG, control group; TKA, total knee arthroplasty; SD, standard deviation; rms, root mean square

	CG		TKΑ baseline		p-value baseline	TKΑ 4 months		p-value 4 months	TKΑ 12 months		p-value 12 months
	mean	SD	mean	SD		mean	SD		mean	SD	
kyphotic angle (°)	56.18	9.37	62.46	13.70	0.074	63.15	15.11	0.065	63.67	12.51	0.023
lordotic angle (°)	42.26	9.13	48.76	16.80	0.109	45.46	14.98	0.382	48.30	17.02	0.139
sagittal imbalance (°)	4.27	2.70	7.57	4.46	0.004	7.89	3.93	0.001	7.70	4.67	0.004
coronal imbalance (°)	1.16	0.69	1.22	1.00	0.819	1.17	1.03	0,957	1.35	1.11	0.481
apical deviation rms (mm)	4.94	1.81	5.94	2.70	0.136	6.05	2.58	0.090	5.31	2.27	0.536
scoliosis angle (°)	12.96	4.45	16.39	5.81	0.026	17.70	5.88	0.003	15.13	4.91	0.115
vertebral rotation rms (mm)	3.98	1.65	4.52	2.58	0.387	5.57	3.10	0.036	5.14	2.57	0.071
trunk torsion (°)	4.16	3.82	7.65	6.46	0.031	8.04	4.78	0.003	7.70	6.03	0.018
pelvic inclination (°)	19.04	8.97	25.43	13.04	0.057	23.17	11.96	0.180	24.74	12.98	0.081
pelvic obliquity (°)	0.96	1.10	2.74	2.78	0.008	1.83	2.44	0.129	1.43	1.73	0.258
pelvic torsion (°)	2.64	1.50	2.57	2.11	0.887	2.43	1.75	0.664	2.04	1.46	0.170
pelvis rotation (°)	2.08	2.48	3.78	4.80	0.125	2.17	3.10	0.908	1.61	2.35	0.504

## Discussion

To our knowledge, this is the first study on truncal changes in patients after THA or TKA operation due to OA using surface topography.

Patients with THA

In the available literature, there are seven radiological studies on truncal changes after THA using other methods, and two studies reported differences in the truncal parameters before and after THA. Peleg Ben-Galim et al. and Radcliff et al. described that spinal measurements after standing lateral radiographs did not differ before and after THA [12,13]. Wen-Jie Weng et al. did not identify significant changes in lumbar and pelvic alignments on lateral radiographs after THA [14]. Bredow et al. described that THA did not influence pelvic position and sagittal alignment using EOS imaging [15]. In the study of Eynazov et al., no significant changes were noted in spinal sagittal radiographic measurements six months after THA [16]. Eguchi et al. described that radiographic parameter lumbar scoliosis significantly reduced patients with unilateral hip osteoarthritis four months after THA [17]. Piazzolla et al. described that radiographic parameters such as lumbar lordosis, thoracic kyphosis, sagittal vertical axis, pelvic tilt, and sacral slope were improved six months after THA for the group of the patients with concomitant low back pain preoperatively. For the group of the patients without low back pain preoperatively, the radiographic parameters did not differ after THA [18].

The results of our study 12 months after THA revealed that the pelvic obliquity was improved to normal levels in the pelvis. Thus, 12 months after the THA, the pelvis was changed in the coronal plane. The correction of the pelvis after THA may be due to mechanical and functional reasons: (a) the correction of hip axis, (b) less flexion-contraction of the hip joint, (c) excision of the joint capsule and the osteophytes, (d) correction of the narrowed joint space, and (e) reduced pain after THA.

In the spine, the kyphotic angle was increased to higher levels after THA and the spine was changed in the sagittal plane. This finding could be attributed to the normal aging of the patients during this study [19-20]. The increased kyphotic angle could also be a compensatory mechanism for the long-term increase (numerically but not statistically significant) of the lordotic angle of the patients after the THA (four months postoperatively 45.74[°] → 12 months postoperatively 47.80[°]). However, it is notable that the kyphotic angle was already increased preoperatively, and hence, the light higher value postoperatively is not clinically significant.

It must be noted that the patients with hip OA exhibited increased values for sagittal imbalance, trunk torsion, and pelvic inclination preoperatively compared with the CG. These results showed that patients with hip OA had more anteverted pelvis and forward inclined spine and coincide with the results of a pertinent study [21]. Postoperatively, the patients continued to exhibit increased values for these parameters compared with the CG. Thus, THA did not improve these parameters for the spine and pelvis.

Patients with TKA

In the available literature, there is only one radiological study on truncal changes after TKA. Lee et al. reported no difference in all measurements except the sacral slope in lateral radiographs of the entire spine [22]. In our study, the Diers Formetric four-D analysis system did not calculate the sacral slope, and hence, the comparison with our study is not applicable.

The results in this study 12 months after TKA revealed that pelvic obliquity was improved to normal levels in the pelvis. Thus, 12 months after the TKA, the pelvis was changed in the coronal plane. Patients who underwent a TKA operation did not have problems with their pelvis. Τhe correction of the pelvic parameter was due to (a) the correction of the knee axis, (b) reduced flexion-contraction of the knee joint, (c) excision of the osteophytes, (d) correction of the narrowed joint space, and (e) reduced pain after TKA.

It is noteworthy that the patients with knee OA exhibited increased values for sagittal imbalance, and trunk torsion postoperatively compared with the CG. These results showed that the patients with knee OA had a significant forward spinal inclination and coincide with the results of the pertinent study [23]. Postoperatively, patients who underwent operation continued to exhibit increased values for these parameters compared with the CG. Thus, TKA did not improve these parameters in the spine. The scoliosis angle was slightly increased in the patients with knee OA compared with the CG, but it changed to normal levels 12 months postoperatively. Thus, improvement of this parameter after TKA may be a result of the improvement of pelvic obliquity.

Effect of THA and TKA

Most studies assess the effect of THA and TKA on sagittal spinal-pelvic-leg alignment. Abnormal alignment causes spinal disorders, whereas normal alignment in the sagittal plane is critical to maintain a right standing position [6,24]. The Diers Formetric four-D analysis system provides a three-D model of the spine and pelvis. Thus, it is the first study that highlights the improvement of the pelvis in the coronal plane after the THA and TKA.

It is very important to note that pelvic obliquity was improved to normal levels 12 months after THA and TKA. This is a novel finding for patients after THA and TKA. Preoperatively, the leg length of the patients was practically equal when measuring the distance between the anterior inferior iliac spine and the lateral malleolus. However, after THA and TKA, the improvement of the pelvic obliquity indicates improvement for the limb length inequality even for patients with almost equal limb length.

Numerous investigations have explored the possible relationships between limb length inequality and orthopedic pathologies. Scoliosis, low back pain, arthritis of the spine, hip osteoarthritis, knee pain, and lower extremity stress fractures have been associated with limb length inequality. Additionally, limb length inequality seemed to influence several functional activities such as walking, running, standing balance and posture, and to cause functional limitations [25-27]. Thus, the improvement of the pelvic obliquity after THA and TKA may be very important in improving patients' everyday function as well as preventing musculoskeletal disorders. Further studies of low back pain, hip and knee pain before and after THA and TKA, and the correlation between them and truncal parameters will be valuable and such studies are already in progress from our team.

Limitations

There were some limitations to this study. The number of patients was limited and some of them failed to have a follow-up. However, this study is ongoing with the recruitment of new patients, and new, more reliable results will be available in the future. Furthermore, the operations were performed by four orthopedic surgeons. However, all of them were experienced and used the same surgical approach and technique.

## Conclusions

THA or TKA not only restores the hip or knee range of motion to a more normal condition and reduces severe pain but also causes truncal morphological alterations. In particular, the pelvis was improved in the coronal plane, and the pelvic obliquity was changed to normal levels after THA or TKA contributed to a correct patient posture and balance.
